# Intraoperative arterial pressure and delayed cerebral ischemia in patients with aneurysmal subarachnoid hemorrhage after surgical clipping: A retrospective cohort study

**DOI:** 10.3389/fnins.2023.1064987

**Published:** 2023-02-17

**Authors:** Jie Wang, Runting Li, Shu Li, Tingting Ma, Xingyue Zhang, Yue Ren, Xiaolin Chen, Yuming Peng

**Affiliations:** ^1^Department of Anesthesiology, Beijing Tiantan Hospital, Capital Medical University, Beijing, China; ^2^Department of Neurosurgery, Beijing Tiantan Hospital, Capital Medical University, Beijing, China; ^3^China National Clinical Research Center for Neurological Diseases, Beijing, China

**Keywords:** aneurysmal subarachnoid hemorrhage, delayed cerebral ischemia, intraoperative arterial pressure, threshold, risk factor

## Abstract

**Background:**

Delayed cerebral ischemia (DCI) is the major predictor of poor outcomes in patients with aSAH. Previous studies have attempted to assess the relationship between controlling blood pressure and DCI. However, the management of intraoperative blood pressure in reducing the occurrence of DCI still remains inconclusive.

**Methods:**

All patients with aSAH who received general anesthesia for surgical clipping between January 2015 and December 2020 were prospectively reviewed. Patients were divided in the DCI group or the non-DCI group depending on whether DCI occurred or not. Intraoperative arterial pressure was measured every minute and recorded in an electronic anesthesia recording system along with intraoperative medication and other vital signs. The initial neurological function score, aneurysm characteristics, surgical and anesthetic information, and outcomes were compared between the DCI and the non-DCI groups.

**Results:**

Among 534 patients who were enrolled, a total of 164 (30.71%) patients experienced DCI. The baseline characteristics of patients were similar between the groups. The World Federation of Neurosurgical Societies (WFNS) Scale > 3, age ≥ 70 years, and the modified Fisher Scale > 2 were significantly higher in patients with DCI than those without. Though it was the second derivative of the regression analysis, 105 mmHg was adopted as the threshold for intraoperative hypotension and was not associated with DCI.

**Conclusions:**

The threshold of 105 mmHg was adopted as intraoperative hypotension even though it was the second derivative of the regression analysis and could not be proved to be associated with delayed cerebral ischemia adjusted by the baseline severity of aSAH and age.

## Introduction

Aneurysmal subarachnoid hemorrhage (aSAH) is a devastating neurological event with a mortality of 30% after 3 months (Etminan et al., 2019; Ganaw et al., 2022). Delayed cerebral ischemia (DCI) is the leading cause of death and disability in patients with aSAH with secured aneurysms and develops within several days (3–21 days post-insult), accounting for up to 30% of new neurological deficits after the initial hemorrhage and leaving the majority of survivors with motor deficits, cognitive dysfunction, and reduced quality of life (Francoeur and Mayer, 2016). The underlying pathophysiological mechanisms of DCI include arterial and arteriole vasospasm, cortical spreading ischemia, and microcirculatory vasoconstriction and thrombosis (de Oliveira Manoel et al., 2016).

In patients with aSAH, blood pressure control initially presents a Scylla and Charybdis dilemma: leaving the patient with untreated high blood pressure increases the risk of rebleeding, but lowering blood pressure increases the risk of DCI (Stienen et al., 2018). The importance of striking a balance between the prevention of rebleeding and the avoidance of secondary cerebral ischemia is highlighted, especially during the initial treatment (Ma and Bebawy, 2022). However, there is disagreement about the target blood pressure threshold (Steiner et al., 2013; Minhas et al., 2022).

Intraoperative cerebral blood flow is mainly determined by cerebral perfusion pressure, which is closely dependent on blood pressure (Dodd et al., 2021). Hypertension, in combination with hemodilution and hypervolemia, is used to prevent potential causes of delayed cerebral ischemia (Treggiari and Deem, 2009). However, the efficacy of hypertension in reducing delayed cerebral ischemia is only based on case series or some small-sample randomized clinical trials (Gathier et al., 2018; Haegens et al., 2018; Esmaeeli et al., 2021), and the conclusion remains elusive.

Therefore, we primarily aimed to investigate the association between intraoperative blood pressure and delayed cerebral ischemia in patients with aneurysmal subarachnoid hemorrhage. We also investigated the impact of intraoperative blood pressure on the clinical outcome at discharge.

## Materials and methods

### Participants

This was a retrospective and single-center cohort study. Patients who underwent emergent surgical clipping after an aSAH under general anesthesia at Beijing Tiantan Hospital, Capital Medical University, between 2015 and 2020 were included in this study. All the neurosurgical data were retrieved from the “Long-term Prognosis of Emergency Aneurysmal Subarachnoid Hemorrhage (LongTEAM)” study. This study was approved by the local institutional ethical review board. The study was registered in ClinicalTrials.gov with the number NCT04785976. Written informed consent for clinical analyses was obtained from the patients or their surrogates. The article adhered to the applicable Strengthening the Reporting of Observational Studies in Epidemiology (STROBE) standards for observational studies.

Adult patients with emergent admission for surgical clipping due to aSAH were enrolled in this study. The initial clinical severity of aSAH was accessed immediately upon admission. SAH was documented by angiography and confirmed by computed tomography or lumbar puncture. The exclusion criteria were as follows: (1) treatment by both surgical clipping and endovascular coiling; (2) other neurological diseases (tumor, vascular malformation, Parkinson's disease, multiple sclerosis, and primary epilepsy); (3) history of neurosurgery prior to rupture; and (4) incomplete perioperative data.

### Data collection and processing

Two independent researchers reviewed the electronic medical records and extracted postoperative data. Differences were resolved by a senior investigator. Baseline data included demographic and morphometric characteristics, American Society of Anesthesiologists physical status, coexisting medical condition, pre-operative medication, and neurological function scales, including the Glasgow Coma Scale, the Hunt and Hess Scale, the World Federation of Neurosurgical Societies Scale, and the modified Rankin Scale, which were assessed based on guidelines (Connolly et al., 2012).

Intraoperative data included the duration of surgery, medication, and fluid input and output. Post-operative delayed tracheal extubation, length of hospital stay, medical cost, and favorable clinical outcome at discharge. Favorable clinical outcomes were defined by the modified Rankin Scale score of 0–2, ranging from 0 (no symptoms) to 6 (dead). Poor clinical outcomes were defined by the mRS score of 3–6.

### Intraoperative blood pressure

Intraoperative blood pressure data were extracted from our Anesthesia Information Management System (AIMS, version 5.0, Wangfeng Mingyue Ltd, China). Most of the patients had arterial catheters inserted into the dorsal pedis or the radial artery with transducers positioned at the level of the right atrium. The invasive blood pressure measurement system included a built-in filter for artifacts that reduced the resonant effects of the tubing system, in which the blood pressure was recorded at 10-s intervals. Non-invasive blood pressure was monitored with a brachial cuff-based oscillometric method at 5-min intervals.

Blood pressure data were excluded using a previously reported method adjusted with our data if (1) invasive data during the initial first 5-min corresponded to the “flushing, leveling and zeroing the transducer” segment; (2) data showed sudden changes of more than 30 mmHg in adjacent invasive pressure values without any annotation identifying clinical causes (Salmasi et al., 2017); (3) invasive data remained unchanged for more than 5 min, which we assumed indicated that the monitoring line was blocked; and (4) data showed values over 300 mmHg or lower than 20 mmHg.

### Delayed cerebral infarction

All patients underwent angiography to diagnose the ruptured aneurysm; patients with a negative angiogram were excluded from the present study. The remaining patients were screened for DIC by computed tomography, and the screening was finally confirmed by MRI. Computed tomography or magnetic resonance imaging was performed postoperatively 24 to 48 h before discharge. In addition, CT perfusion (CTP) or transcranial Doppler (TCD) may also be used.

Delayed cerebral ischemia (DCI) was defined as the development of new focal neurological deficits or a decrease in ≥2 points on the Glasgow Coma Scale score for a duration ≥1 h in conscious patients, with the exclusion of any other explanation for the deterioration, such as infection with an associated decrease in consciousness level, rebleeding, edema (increasing) hydrocephalus, hypoglycemia or hyponatremia or hypotension, or any other possible cause for deterioration as judged by the treating physician (Vergouwen et al., 2010; Fan et al., 2021). Alternatively, new ischemic lesions were found by CT and/or MRI, or perfusion decreased in the corresponding brain functional area showed by CTP, and/or blood flow velocity increased in the corresponding brain area showed by TCD.

### Statistical analysis

Patients were analyzed as to whether they experienced DCI before discharge. Categorical data are presented as count (percentage) and analyzed using a two-tailed chi-square test with continuity correction or the Fisher exact test. The continuous variable is presented as the mean and standard deviation (SD) or the median and inter-quartile range (IQR) and compared with Student's *t*-test if normally distributed or the Mann–Whitney test if not. The mean difference or relative risks with a 95% confidence interval were calculated for a comparison of continuous data and categorical data.

We used logistic regression to identify change points that are indicative of a harm threshold as reported by previous researchers (Salmasi et al., 2017). We de-noised the data using a simple moving average method with a duration of 5 min and determined the lowest blood pressure for each subject. We then calculated the cumulative duration of the lowest MAP for each subject and depicted the estimated probability of DCI in patients with a cumulative duration of 5–10 min, which was one of the most common durations of the lowest blood pressure using logistic regression, with predictive margins of the probability of DCI for MAP from 40 to 180 mmHg. The relationship between the lowest sustained pressure and DCI was evaluated using logistic regression. The inflection point (potential threshold) was identified from the second derivative of the regression using the “Kneedle” method programmed in Python (Satopaa et al., 2011).

The area under harm thresholds was defined as the cumulative sums of the area using the trapezoid rule and is reported in units of mmHg times minutes. Calculation of the area under harm thresholds with specific threshold started and ended when MAP was below and greater than this threshold. The duration of hypotension was calculated as the cumulative duration below each threshold, in minutes. The time-weighted average MAP was derived by dividing the area below the threshold by the duration of anesthesia (Salmasi et al., 2017; Schacham et al., 2022).

The results are presented as odds ratios (OR) or adjusted odds ratios (aOR) with 95% confidence intervals (CIs). Model diagnostics were also reported, including the Hosmer–Lemeshow goodness-of-fit test and the area under the receiver operating characteristic curve. The interaction was detected using interaction terms in the regression model, and a variance inflation factor of < 5 was considered as an absence of multicollinearity. For all outcomes, a *P* < 0.05 was considered statistically significant. All statistical analyses were performed using the Stata/SE 16.0 (StataCorp, TX, USA).

## Results

**Figure 1:**
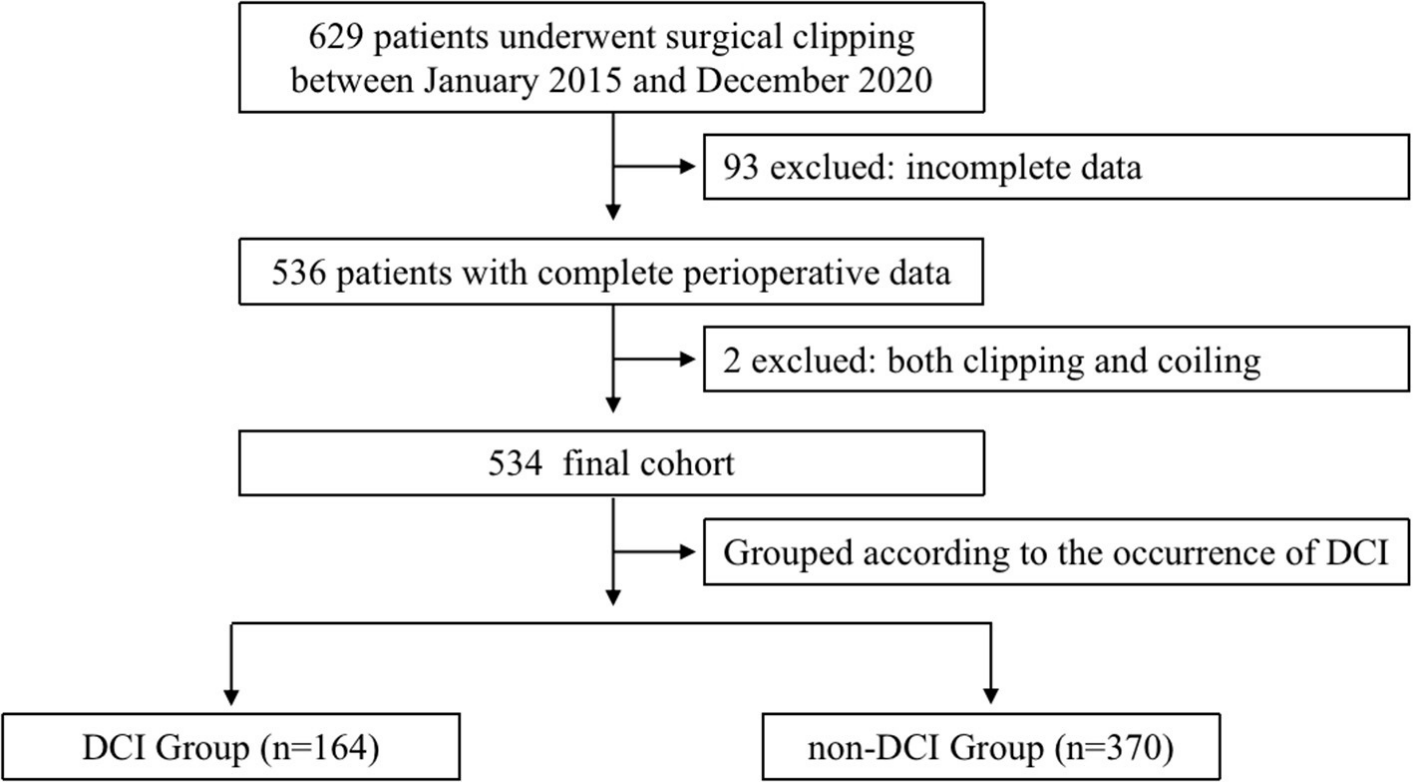
Flowchart. The chart shows the inclusion and exclusion criteria in the study. DCI indicates delayed cerebral ischemia.

Of the 629 patients who underwent emergent surgical clipping between January 2015 and December 2020, 534 patients were enrolled (Figure 1). A total of 164 (30.7%) patients experienced DCI, and the remaining 370 patients did not experience DCI. Between patients who experienced DCI and those who did not, no significant differences in demographics, past medical history, medication, ASA physical status, OAA/S scale, or preoperative Hunt–Hess grading were found (Table 1). However, the percentage of patients with age over 70 years (10.4 vs. 4.3%, *P* < 0.05), a modified Fisher Scale score of 3 to 4 (83.5 vs. 71.1%, *P* < 0.05), and the WFNS Scale of Grade 4 to 5 (29.3 vs. 18.7%, *P* < 0.05) was significantly higher in patients with DCI than those without. Aneurysm characteristics including whether there are multiple aneurysms, the size of the aneurysm, and the locations of ruptured aneurysms did not significantly differ between groups.

**Table 1:** Characteristics of the patients at baseline.

	**All patients**	**DCI**	**Without DCI**	**P-value**
	***n*** = **534**	***n*** = **164**	***n*** = **370**	
Mean age (SD), years	54.7 (10.3)	55.4 (10.5)	54.4 (10.1)	0.330
Age > 70, no. (%)	33 (6.18)	17 (10.4)	16 (4.3)	0.007^*^
Male sex, no. (%)	225 (42.1)	69 (42.1)	156 (42.2)	0.985
Mean BMI (STD)	25.0 (3.9)	25.2 (4.0)	25.0 (3.8)	0.535
**Medical history, no. (%)**				
Drinking	108 (20.2)	35 (21.3)	73 (19.7)	0.669
Smoking	150 (28.1)	44 (26.8)	106 (28.6)	0.666
Pulmonary disease	65 (12.2)	23 (14)	42 (11.4)	0.334
Hyperlipidemia	16 (3.0)	5 (3.1)	11 (3.0)	0.962
Stroke	33 (6.2)	12 (7.3)	21 (5.7)	0.467
Arrythmia	12 (2.2)	6 (3.7)	6 (1.6)	0.143
Diabetes	30 (5.6)	12 (7.3)	18 (4.9)	0.256
Hypertension	298 (55.8)	94 (57.3)	204 (55.1)	0.640
**Preoperative medication**				
Aspiration, no. (%)	10 (1.9)	3 (1.8)	7 (1.9)	0.961
Endotreal intubation, no. (%)	20 (3.8)	10 (6.1)	10 (2.7)	0.057
Electrolyte disorder, no. (%)	25 (4.7)	9 (5.5)	16 (4.3)	0.557
Antiplatelet, no. (%)	31 (5.8)	10 (6.1)	21 (5.7)	0.847
Antihypertensives treatment, no. (%)	208 (39)	61 (37.2)	147 (39.7)	0.580
**Pre-operative assessments**				
**ASA physical status, no. (%)**				**0.447**
II–III	491 (92.0)	153 (93.3)	338 (91.4)	
IV–V	43 (8.1)	11 (6.7)	32 (8.7)	
**OAA/S scale, no. (%)**				**0.851**
≤ 2	47 (8.8)	15 (9.2)	32 (8.7)	
>2	487 (91.2)	149 (90.9)	338 (91.4)	
**Hunt-Hess, no. (%)**				**0.464**
Grade 1–3	492 (92.1)	149 (90.9)	343 (92.7)	
Grade 4–5	42 (7.8)	15 (9.2)	27 (7.3)	
**Modified fisher scale, no. (%)**				<0.002^*^
Grade 0–2	134 (25.1)	27 (16.5)	107 (28.9)	
Grade 3–4	400 (74.9)	137 (83.5)	263 (71.1)	
**WFNS scale, no. (%)**				**0.006** ^*^
Grade 1–3	417 (78.1)	116 (70.7)	301 (81.4)	
Grades 4–5	117 (21.9)	48 (29.3)	69 (18.7)	
**Aneurysm characteristics**				
Multiple, no. (%)	102 (19.0)	31 (18.9)	71 (19.1)	0.938
Mean diameter (IQR)	7.3 (4.4)	7.5 (4.5)	7.3 (4.4)	0.557
Mean neck diameter (IQR)	4.6 (2.6)	4.7 (2.8)	4.5 (2.6)	0.359
Mean day of treatment (SD)	3.9 (3.0)	3.6 (2.8)	4.0 (3.0)	0.202
**Locations of ruptured aneurysm, no. (%)** ^#^				**0.085**
Anterior circulation	517 (96.8)	162 (98.8)	355 (96.0)	
Posterior circulation	17 (3.2)	2 (1.2)	15 (4.1)	

The independent sample t-test was performed for continuous variables and the chi-square test was performed for categorical variables.

* P-value reflects the statistical comparison between two groups.

^#^Anterior circulation indicated an aneurysm at the anterior cerebral artery, the middle cerebral artery, the anterior communicating artery, the anterior choroidal artery, the internal carotid artery, the ophthalmic artery, and the posterior communicating artery. Posterior circulation indicated an aneurysm at the posterior cerebral artery, the superior cerebellar artery, and the posterior inferior cerebellar artery.

ASA, American Society of Anesthesiology; BMI, body mass index; CT, computed tomography; WFNS, World Federation of Neurosurgical Societies Scale; OAA/S, Observer Assessment of Alertness/Sedation Scale; IQR, inter-quartile range.

Intraoperative anesthetic-related parameters were compared (Table 2). The mean arterial pressure of the patient before intubation in the DCI group and without the DCI group was 108 (14) vs. 111 (27) mmHg, respectively, which was not statistically different. The duration under different intraoperative blood pressure thresholds (110, 105, and 100 mmHg) between the two groups was compared, and no significant difference was found. The type of anesthesia, intraoperative medication, fluid intake and output, and the results of blood gas analysis were comparable between the two groups.

**Table 2:** Surgical and anesthetic-related parameters.

	**All patients**	**DCI**	**Without DCI**	**P-value**
	**(*****n*** = **534)**	**(*****n*** = **164)**	**(*****n*** = **370)**	
**Intraoperative**				
Mean pre-induction MAP, mmHg	110 (23)	108(14)	111 (27)	0.188
Hybriding surgery, no. (%)	66 (12.4)	16 (9.8)	50 (13.5)	0.224
**Type of anesthesia, no. (%)**				**0.672**
TIVA	91 (17.0)	30 (18.3)	61 (16.5)	
Inhalation	7 (1.3)	3 (1.8)	4 (1.1)	
Combined	436 (81.7)	131 (79.9)	305 (82.4)	
Vasopressor, no. (%)	104 (19.5)	33 (20.1)	71 (19.2)	0.802
Mannitol, no. (%)	245 (45.9)	83 (50.6)	162 (43.8)	0.144
Median total input (IQR), ml	2,500 (2,000–3,000)	2,500 (2,000–3,000)	2,500 (2,000–3,000)	0.755
Median urine (IQR), ml	800 (600–1,200)	800 (500–1,200)	800 (600–1,200)	0.491
Median bleed loss (IQR), ml	200 (200–400)	200 (200–400)	200 (150–400)	0.11
**Median duration (IQR), min**				
Surgery	195 (158–243)	199 (166–250)	193 (156–238)	0.182
Anesthesia	296 (255–351)	303 (255−367)	290 (252–345)	0.098
**Blood gas analysis**				
Mean hct (SD), %	34.17 (6.10)	33.54 (6.41)	32.99 (5.98)	0.554
Mean blood glucose (SD), mg/dl	6.526 (1.35)	6.46 (1.14)	6.56 (1.45)	0.622
Mean PaCO_2_ (SD), mmHg	35.587 (4.83)	36.34 (5.19)	35.22 (4.65)	0.124
**Post-operative**				
Delayed tracheal extubation, no. (%)^†^	145 (27.2)	60 (36.6)	85 (23.0)	0.001^*^
Median LOS (IQR), d	14 (10–18)	16 (12–21)	14 (10–17)	0.001^*^
Median Hospital costs (IQR), × 10^3^ RMB	80.1 (66.8–105.7)	92.8 (72.8–126.0)	77.7 (64.3–96.7)	< 0.0001^*^
Good outcome at discharge, no (%)	282 (52.8)	44 (26.8)	228 (64.3)	< 0.0001^*^

The independent sample t-test was performed for continuous variables, and the chi-square test was performed for categorical variables.

* P-value reflects the statistical comparison between two groups.

† Delayed tracheal extubation was defined as the patient not being extubated at the end of the surgical case, before leaving the operating room. MAP, mean arterial pressure; TIVA, total intravenous anesthesia; Hct, hematocrit; PH, potential of hydrogen; PaCO2, pressure of carbon dioxide; LOS, length of hospital stay; IQR, inter-quartile range.

Patients who experienced DCI had a significantly higher proportion of delayed tracheal intubation (36.6 vs. 23.0%, *P* = 0.001), extended hospital stay (17 vs. 15 days, *P* < 0.0001), and higher medical cost (104.6 vs. 84.9 × 10^3^ RMB, *P* < 0.0001). In addition, patients who experienced DCI had a lower incidence of a favorable outcome at discharge (26.8 vs. 64.3%, *P* < 0.0001, Table 2).

**Figure 2:**
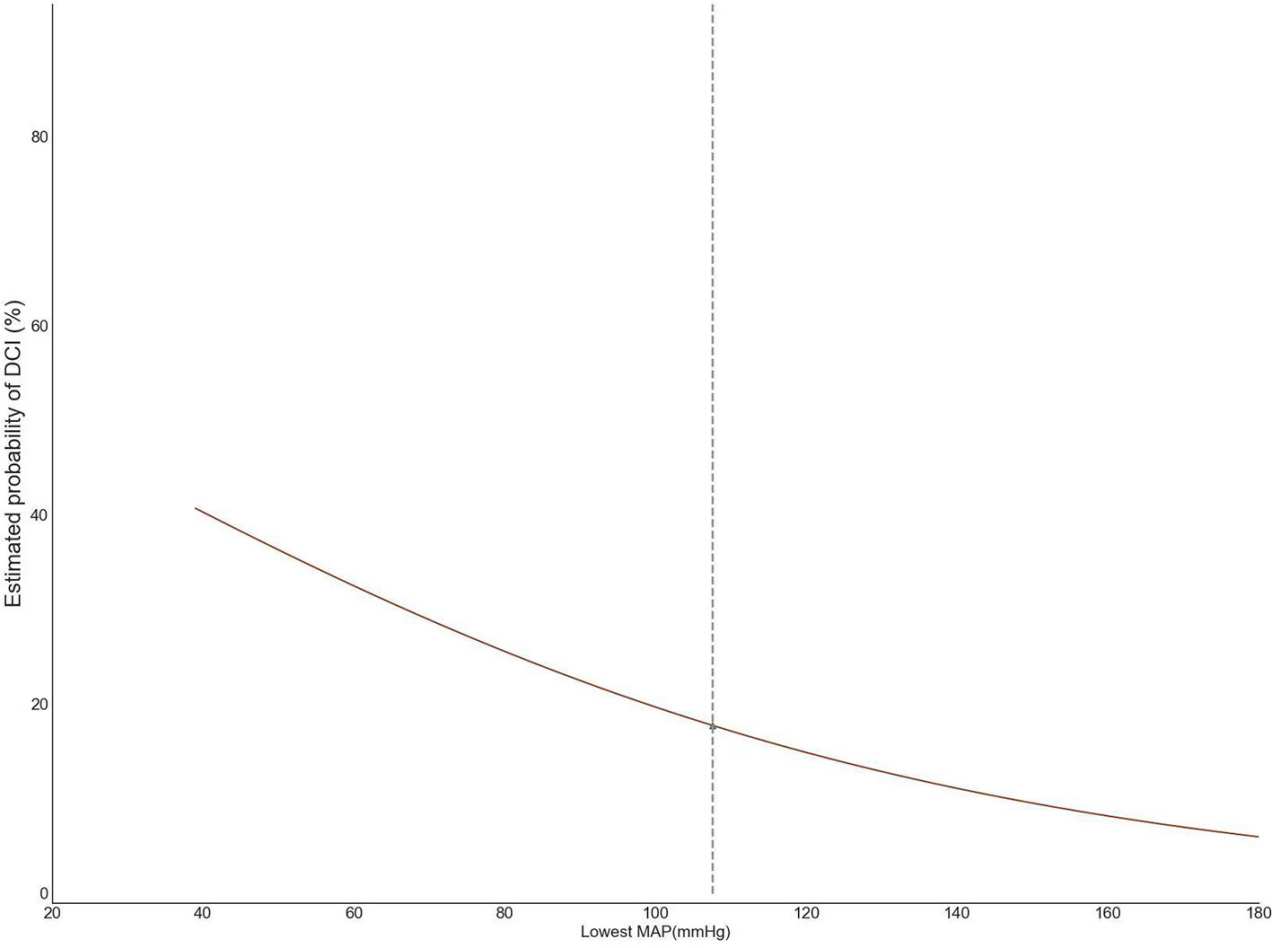
The lowest mean arterial pressure (MAP) thresholds for delayed cerebral ischemia (DCI).

Based on the second derivative for the cut-point of 105 mmHg, we, therefore, adopted 105 mmHg as the threshold for intraoperative hypotension (Figure 2). We further analyzed the area under the receiver operating characteristic and time-weighted average MAP under three thresholds. However, no difference in the median duration of MAP lower than 105 mmHg (236 vs. 228 min, *P* = 0.125), time-weighted average MAP (19.1 vs. 18.8 mmHg/min, *P* = 0.685), or AUC–MAP (5,666 vs. 5,493 mmHg^*^min, *P* = 0.291) between patients with DCI and those without DCI was found. The same trend existed with the other two thresholds (Table 3).

**Table 3:** Hypotension characteristics for various thresholds.

**Characteristics**	**DCI**	**Without DCI**	**P-value**
	***n*** = **164**	***n*** = **370**	
**AUC–MAP (IQR), mmHg** *min			
Below 100 mmHg	4,419 (3,344–6,152)	4,255 (2,601–6,261)	0.341
Below 105 mmHg	5,666 (4,359–7,572)	5,493 (3,631–7,599)	0.291
Below 110 mmHg	6,899 (5,327–9,103)	6,552 (4,600–8,980)	0.263
**Duration (IQR), min**			
Below 100 mmHg	224 (182–273)	214 (174–268)	0.132
Below 105 mmHg	236 (196–285)	228 (188–281)	0.160
Below 110 mmHg	247 (204–300)	239 (198–288)	0.207
**Time-weighted average MAP, mmHg / min**			
Below 100 mmHg	15.4 (10.4–19.5)	14.8 (9.7–20.3)	0.701
Below 105 mmHg	19.1 (14.2–23.7)	18.8 (13.1–24.5)	0.685
Below 110 mmHg	23.1 (18.3–28.1)	23.0 (16.8–28.8)	0.706

AUC, area under curve; MAP, mean arterial pressure; IQR, inter-quartile range.

Variables with statistical differences in univariate analysis were included in multivariate regression analysis and the risk prediction model. We analyzed the adjusted association between intraoperative hypotension and postoperative DCI by age of over 70 years and by aSAH severity in univariate first and then in multivariate analysis. MAP below 105 mmHg for every 30 min did not significantly increase the odds of DCI (odds ratio=1.05, 95% CI: 0.98–1.13) (Table 4) adjusted by the other independent risk factors. The area under receiver operating characteristic (ROC) curve was 0.625 (95%CI: 0.57, 0.68) and *P* = 0.360 in the Hosmer–Lemeshow test. No interaction or multicollinearity was observed in the multivariate model.

**Table 4:** Univariate analysis and multivariate analysis of the association between intraoperative hypotension and DCI.

**Predictor variables**	**Univariate analysis**		**Multivariate analysis**	
	**odds ratio**	**95% CI**	**Adjusted odds ratio**	**95% CI**
WFNS scale > 3	1.81	1.18–2.76	1.68	1.09–2.62
Age ≥ 70 years	2.56	1.26–5.20	2.56	1.24–5.27
mFS score > 2	2.06	1.29–3.30	1.82	1.13–2.95
MAP below 105 mmHg for every 30 min	1.05	0.98–1.12	1.05	0.98–1.13

The univariate analysis and multivariate analysis for DCI were performed with candidate predictor variables. The area under the receiver operating characteristic (ROC) curve was 0.625 (95% CI 0.574, 0.676) and P = 0.360 in the Hosmer–Lemeshow test. No interaction or multicollinearity was observed in the multivariate model. CI, confidence interval; DCI, delayed cerebral ischemia; WFNS, World Federation of Neurosurgical Societies Scale; mFS, modified Fisher Scale.

## Discussion

In the current study, we included 534 patients who underwent surgical clipping after aneurysmal subarachnoid hemorrhage, out of whom 164 (30.7%) patients showed delayed cerebral ischemia. The duration of intraoperative MAP lower than different thresholds was almost similar in patients who had DCI or not. Furthermore, MAP below 105 mmHg for 30 min did not significantly associate with DCI adjusted by the baseline severity of aSAH and age.

In the present study, the incidence of delayed cerebral ischemia was 30.7% (164 out of 524) after surgical clipping ruptured aneurysm, consistent with those of previous studies (Lawton and Vates, 2017; Macdonald and Schweizer, 2017; Anetsberger et al., 2020). The brain after aSAH might undergo cerebral edema, blood–brain barrier disruption, sympathetic nervous system activation, autoregulatory failure, microthrombosis, and inflammation, which might have the potential to lead to the development of DCI ultimately (Lawton and Vates, 2017; Topkoru et al., 2017). In addition, DCI would lead to physical disability and cognitive impairment, which are the major causes of a poor prognosis. In the present study, DCI significantly prolonged the time of endotracheal intubation, duration of hospital stay, worsened neurological function, and increased medical costs.

Maintaining adequate blood pressure is essential for patients under general anesthesia to treat the aneurysm, avoiding hypotension-related cerebral ischemia (Suarez et al., 2006; Pasternak and Lanier, 2015). Induced hypotension in patients with aSAH undergoing surgical clipping was associated with poor neurological outcomes (Mahaney et al., 2012; Ayling et al., 2015). From the triple-H therapy (hypertension, hypervolemia, and hemodilution) for the prevention of DCI (Treggiari et al., 2003) in some small studies (Chang et al., 2000; Hoff et al., 2008), the association between blood pressure and delayed cerebral ischemia remains elusive. A study of 164 aSAH patients receiving surgical clipping suggested that a decrease in blood pressure of more than 50% was associated with a poor outcome. However, the association was no longer significant after adjusting the selection bias (Rosen and Macdonald, 2005; Hoff et al., 2008). A study on 84 patients found a comparable association for intraoperative hypotension [defined as a systolic blood pressure (SBP) <90 mmHg for more than 15 min] (Chang et al., 2000). Another study of 398 patients with aSAH indicated intraoperative hypotension (defined intraoperative hypotension as a reduction of 30 mmHg or at least 20% of the initial SBP, for at least 15 min) as an independent risk factor for the development of a postoperative cerebral infarction (odds ratio, 3.02; 95% CI, 1.29–7.08) (Chong et al., 2008). A prospective observational cohort study from Brazil found that rescue treatment of hypertension induction (HI) and inotropic drugs improved the clinical response and perfusion on CT in patients who suffered secondary ischemic symptoms or neurological deficits after aneurysm occlusion (Steiger et al., 2022). In this retrospective cohort study, the change rate of the estimated probability of DCI tends to remain unchanged as the second derivative approaches zero at the lowest MAP at 105 mmHg; therefore, we adopted 105 mmHg as the threshold for intraoperative hypotension. However, the current study could not examine the effects of hypotension derived by the mathematics calculation (nearly the same blood pressure as the baseline) on the DCI. The present study found no association between intraoperative hypertension and DCI after the clipping of a ruptured aneurysm. On the other hand, the automatic regulation of cerebral blood flow is impaired after aSAH. CBF autoregulation can be divided into static regulation and dynamic regulation. Dynamic CBF autoregulation reflects the instantaneous change in cerebral blood flow when blood pressure fluctuates. Studies showed that dynamic cerebral blood flow autoregulation parameters can be used as objective evaluation indicators for patients with aSAH to identify and predict the high-risk population prone to cerebral canal spasm and delayed cerebral ischemia after onset (Otite et al., 2014). In this study, we did not include CBF autoregulation in patients with aSAH, which may have contributed to the negative results. After the occurrence of aSAH, patients experience drastic physiological changes, and each patient has a different response and adjustment ability to these drastic changes, so the prediction of DCI may require more observation indicators with more detailed dimensions. These results may suggest that the brain is more sensitive to intraoperative hypotension than the myocardium and the kidney (Salmasi et al., 2017). Though the AUC, duration, and time-weighted average MAP below 105 mmHg were similar between patients with DCI and patients without DCI, we should be especially vigilant about hypotension-induced DCI in older patients with aSAH with a higher modified Fisher score and a higher WFNS Scale score.

Lowering blood pressure may attenuate hematoma progression and hemorrhagic transformation in patients with aSAH; in contrast, increased blood pressure could avoid secondary cerebral ischemic damage related to hypotension (Akkermans et al., 2019). In uncontrolled studies, hypertension seems to be more effective in increasing CBF than hemodilution or hypervolemia (Dankbaar et al., 2010). However, in the current study, we did not find a difference in total fluid input volume or hematocrit.

The study has several limitations. First, the information on intraoperative blood pressure was not prospectively collected, which might lead to a measurement bias. Second, we did not perform a pre-estimation of the sample size, and the relatively limited sample size might contribute to the negative association between the blood pressure threshold and DCI. Third, brain CT was not routinely performed, leading to the exclusion of those patients for whom it was uncertain if the primary endpoint, cerebral infarction, was reached. Finally, we did not relate the MAP threshold to the surgical procedure, that is, the target blood pressure for maintaining cerebral perfusion pressure differs before clipping and after clipping. Weiss et al. (2022) found that, at the time of DCI, CPP was significantly lower in patients with DCI, which was due to a significantly lower MAP, whereas ICP was stable before DCI. However, the same study showed that SBP > 180 mmHg may lead to hyperperfusion, presenting as the induced CPP exceeding the calculated optimal CPP significantly. A population selection bias was noticed in this study as most of the patients who came to the hospital were critically ill.

In summary, the presence of DCI was diagnosed in ~30% of the patients with SAH with aneurysm rupture, and baseline SAH severity and age over 70 years old were independent risk factors. Although an association between intraoperative hypotension and the occurrence of postoperative DCI was not found, further prospective research is worth considering with a trial of hypotension avoidance in patients with aSAH.

## Data availability statement

The original contributions presented in the study are included in the article/supplementary material, further inquiries can be directed to the corresponding authors.

## Ethics statement

The studies involving human participants were reviewed and approved by Beijing Tiantan Hospital, Capital Medical University. Written informed consent to participate in this study was provided by the participants' legal guardian/next of kin.

## Author contributions

JW, RL, SL, TM, XZ, and YR performed material preparation, data collection, and analysis. JW wrote the first draft of the manuscript. YP and XC designed the research project and revised the manuscript. All authors contributed to the study's conception and design, commented on previous versions of the manuscript, read, and approved the final manuscript.
